# Economic Analysis of a Multi-Sided Platform for Sensor-Based Services in the Internet of Things

**DOI:** 10.3390/s19020373

**Published:** 2019-01-17

**Authors:** Luis Guijarro, Jose R. Vidal, Vicent Pla, Maurizio Naldi

**Affiliations:** 1 Instituto ITACA, Universitat Politècnica de València, 46020 Valencia, Spain; jrvidal@dcom.upv.es (J.R.V.); vpla@upv.es (V.P.); 2 Dipartimento di Ingegneria Civile e Ingegneria Informatica, Università di Roma Tor Vergata, 00133 Rome, Italy; maurizio.naldi@uniroma2.it

**Keywords:** sensor networks, Internet of Things, network economics, platform, multi-sided markets, pricing

## Abstract

A business model for sensor-based services is proposed where a platform creates a multi-sided market. The business model comprises a platform that serves as an intermediary between human users, app developers, and sensor networks, so that the users use the apps and the apps process the data supplied by the sensor networks. The platform, acting as a monopolist, posts a fee for each of the three sides so as to maximize its profit. This business model intends to mimic the market-creating innovation that main mobile apps platforms have generated in the smartphone sector. We conduct an analysis of the profit maximization problem faced by the platform, show that optimum prices exist for any parameter value, and show that these prices always induce an equilibrium in the number of agents from each side that join the platform. We show that the relative strength of the value that advertisers attach to the users determines the platform price structure. Depending on the value of this relative strength, two alternative subsidizing strategies are feasible: to subsidize either the users’ subscription or the developers’ registration. Finally, all agents benefit from an increase in the population at any of the three sides. This result provides a rationale for incentivizing not only the user participation, but also the entry of developer undertakings and the deployment of wireless sensor network infrastructure.

## 1. Introduction

The Internet of Things (IoT) is one of the hottest topics being debated today across industries worldwide. Although IoT devices are becoming omnipresent, the fact is that the market for services related to these devices is immature. Bohli, Sorge and Westhoff [1] stated that the commercial success of sensor-based services requires that both the appropriate market structure and corresponding pricing schemes be well understood. Among the different structures or business models that may be implemented for sensor-based services, some authors claim that the “platform” business model (also known as multi-sided market), with which companies such as Apple, Google, or Amazon have succeeded, is readily transferable to the Internet of Things [2].

This work proposes such a business model and analyzes the provision of sensor-based services by a platform that creates a multi-sided market.

### 1.1. IoT and Multi-Sided Markets

There have been various attempts to define the concept of a multi-sided market. We present below the most significant ones.

Armstrong [3] defined multi-sided markets as “markets in which two or more groups of agents interact via intermediaries or platforms. Surplus is created—or destroyed in the case of negative externalities—when the groups interact. […] In a set of interesting cases, cross-group externalities are present, and the benefit enjoyed by a member of one group depends upon how well the platform does in attracting customers from the other group.”

Rochet and Tirole [4] roughly defined multi-sided markets as “markets in which one or several platforms enable interactions between end-users and try to get the two (or multiple) sides ‘on board’ by appropriately charging each side.” However, they go further and define “a two-sided market as one in which the volume of transactions between end-users depends on the structure and not only on the overall level of the fees charged by the platform.”

Finally, Hagiu and Wright [5] believed that “At the most fundamental level, MSPs [multi-sided platforms] have two key features beyond any other requirements (such as indirect network effects [referring to [3]] or non-neutrality of fees [referring to [4]]). They enable direct interactions between two or more distinct sides. [And] each side is affiliated with the platform.”

Video game platforms and operating systems, first, and mobile apps, more recently, are examples of two-sided platforms, and they have been very successfully implemented. Based on the observation of the mobile apps market, Schuermans and Vakulenko [2] argue that “the evolution in mobile in the past six years holds a clear lesson for the Internet of Things. To realize its full potential, the fledgling Internet of Things industry needs to follow iOS’s and Android’s recipe of market-creating innovation.” According to the authors, “iOS in particular derives a significant part of its brand value and pricing power from its reputation to have most innovative apps coming to iOS first.” Furthermore, having witnessed that “the same steady stream of developer-driven innovation is already emerging in IoT,” they argued that “wide-ranging and often unexpected devices, services and apps that come from a growing community of Internet of Things developers is the main factor that will drive demand for IoT to unseen heights.” Finally, in order to make it possible, “instead of creating applications around specific devices (a parking app using parking sensors, a mobile app to unlock my door, and so on), data from all kinds of inputs can be gathered on a central platform.” In other words, the platform should focus on supplying data and providing support to third-party applications.

### 1.2. Objectives, Related Work and Structure

Motivated by the discussion by Schuermans and Vakulenko [2], this paper proposes a business model where a platform creates a three-sided market. Aligned with the above discussion on multi-sided markets:

- The platform will intermediate between the three groups of agents that interact in an IoT market: users, application developers, and data sensors. By incorporating the data sensors, as stated above, the platform will no longer be focused on developing specific-purpose apps, but on gathering relevant data for the app developers, instead.
- The platform will set corresponding fees/payments for each side or group in order to maximize the platform’s profits.
- The profit maximizing fees/payments will incentivize the affiliation of the different groups leveraging the effect that each side has on the other sides (cross externalities).

In this work, we identify and characterize the relevant cross externalities between the three groups and propose a fee structure for the platform. Our aim is to check whether a multi-sided market emerges, in the sense explained in Section 1.1, and to analyze the influence of the different parameters on the profits, affiliations, and welfare of each group of agents on the equilibrium.

The contributions of the paper are then:

- We propose a business model for a platform that intermediates in a three-sided IoT market.
- We identify and characterize the relevant cross externalities between the three sides and propose a fee structure for the platform.
- We derive the influence of the most relevant parameters on the profits, affiliations, and welfare of each group of agents on the equilibrium. These parameters are the relative strength attached by the advertisers to the subscribers and the population size of the groups.

There are some works that have analyzed multi-sided markets operated by platforms, in contexts such as crowdfunding [6], smartphones [7], and video games [8]. However, few papers have studied this market structure in the context of telecommunications services, e.g., see [9] for an application of this market structure to Internet access provision, in order to evaluate the issue of network neutrality. To the best of our knowledge, this paper is the first to model analytically a multi-sided market within an IoT context, progressing beyond a qualitative appraisal of such sorts of models.

The authors researched previously the topic of sensor-based services [10] and specifically of multi-sided markets operated by platforms in the context of IoT. In [11,12], a two-sided market was modeled, and alternative pricing schemes were comparatively analyzed. In [13], a preliminary model of an IoT market was presented. The current work extends this preliminary analysis in two ways: it conducts a complete equilibrium and optimization analysis, and it discusses an extended array of results that produce relevant conclusions.

The paper is structured as follows. In the next section, the business model is described, as well as the specific model for each agent, including the payment flow, the modeling decisions, and assumptions made, e.g., the utility expressions used in the analysis. In Section 3, the profit maximizing prices and equilibrium values are derived. In Section 4, we present and discuss some numerical results to illustrate the main characteristics of the model. Finally, Section 5 draws some conclusions.

## 2. Model Description

**Figure 1:**
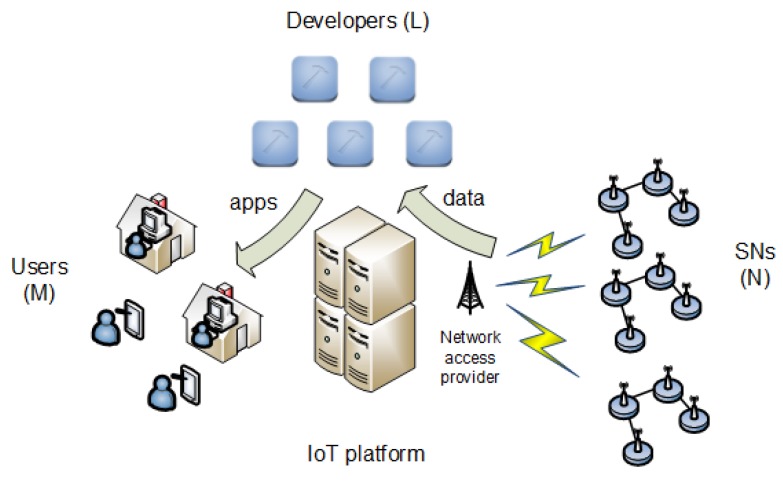
Scenario. SNs, sensor networks.

The scenario modeled in this work is shown in Figure 1. It comprises

- *N* sensor networks (SNs)
- one IoT platform
- *L* developers
- *M* users

**Figure 2:**
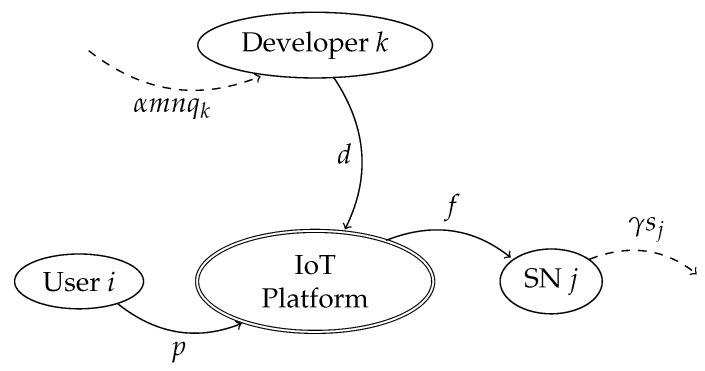
Platform payment flow model.

Basically, the platform gathers the data that are sensed by the SNs, and the developers make use of the data for composing apps. The platform also provides the means to make these apps available for the users. We assume that the platform sets the business on a preexisting ecosystem of SNs and developers, both of which undertake their respective economic activities (e.g., data sensing for the SNs and app development for the developers). The payment flows for all agents in the scenario are detailed in Figure 2 and are explained in the following subsections.

We therefore assume a monopoly for the market structure. The primary reason is that our priority in this work is the assessment of the feasibility of the business model described in this section for the different stakeholders (users, developers, SNs, and the platform) and the analysis of the influence of the different parameters on this feasibility. We defer the analysis of an oligopoly to future works. Still, we can envision a scenario where barriers to entry are erected by the platform, e.g., a platform with a dominant position in the mobile application market may implement different fidelity schemes to entice the users and the developers to join the platform in the adjacent market of the apps for the IoT. Alternatively, the incumbent platform can engage in limit pricing in order to deter entry by taking advantage of an entry cost borne by the potential entrant platform. Under this situation, the incumbent would have the option to either accommodate entry (resulting in a duopoly) or exclude entry and enjoy monopolistic (but not maximum) profits. Entry exclusion will be such that the incumbent’s prices must be low enough to not leave any room for the entrant to cover its fixed cost of entry. On the basis of this example, we argue that the platform is a monopoly of the sort of an unregulated very large firm, so that depending on the results of the analysis, regulatory measures should be adopted on the basis of the improvement of the social welfare.

A summary of the notation used in this paper is given in Table 1.

### 2.1. Sensor Networks

There exist *N* sensor network (SN) islands. Each SN island consists of a sensor infrastructure that has been deployed by an undertaking. This undertaking may be a private enterprise or a public project; in either case, each undertaking performs an activity that justifies the infrastructure deployment. As far as our business model is concerned, each SN senses data as part of the main activity of the undertaking and may decide to generate a data stream as a sub-product of this main activity. These data may be supplied to the platform, allowing the SN to get additional revenue and thus providing a complementary way to monetize the data. In terms of costs, an investment sunk cost has already been incurred, and it is not relevant to our analysis. In terms of ownership, the data are owned by the undertaking, and the supply to the platform can be performed by an ownership transfer or a license agreement. This approach has been adopted by the authors in previous research [10,12], but also by other researchers [14].

Each SN is then capable of generating a stream of data for the platform and is paid a fixed amount *f* by the platform. This payment *f* is the same for all SNs that decide to supply data to the platform. Note that we are anticipating that the platform will not charge the SNs, but will pay them a fee.

As regards the costs, only incremental costs incurred by the data supply to the platform are relevant for our analysis. SN *j* pays a network access fee to the network access provider, which depends on its upload requirements, which may be stated in terms of data rate, for instance. We denote SN *j*’s upload requirements as $s_{j}$, and the network access fee paid by SN *j* will be by $\gamma s_{j}$, where γ is then a price per upload capacity unit. We assume that the number of SNs is sufficiently high so that each is assumed to take prices γ and *f* as given.

We then have a set of *N* SNs with different upload requirements *s*. We model the heterogeneity of the SNs in terms of $s_{j}$ through a random variable S uniformly distributed in the interval $\left[0,1\right]$.

SN *j* obtains a profit equal to the revenue *f* minus the cost $\gamma s_{j}$ if it joins the platform or a profit equal to zero if it does not. We denote the profit by ${\hat{\Pi }}_{j}^{s}\equiv f-\gamma s_{j}$. We will use superscript *s* to denote a quantity related to the SNs and superscript *d* to denote the developers.Thus, the profit of SN *j* is:

(1) $$\Pi_{j}^{s}=\max\left\{0,{\hat{\Pi }}_{j}^{s}\right\},$$

and it will join the platform if, and only if, ${\hat{\Pi }}_{j}^{s}\geq 0$; the probability of this event is:

(2) $$P\left(f-\gamma S\geq 0\right)=P\left(S\leq \frac{f}{\gamma }\right).$$

The number of connected SNs N (i.e., the SNs that join the platform) is then a random variable, and its expected value, *n*, is given by:

(3) $$n=E\;\left[N\right]=NP\left(S\leq \frac{f}{\gamma }\right).$$

Finally, the SNs’ welfare NW is defined as the average aggregate profits of the SNs, i.e.,

(4) $$\mathrm{NW}\equiv NE\;\left[\Pi^{s}\right]=N\int_{0}^{1}\max\left\{0,f-\gamma s\right\}\;ds.$$

which provides a single measure of the overall benefit received by the SNs.

### 2.2. App Developers

There are *L* app developers that may access the data gathered by the platform in order to compose the apps, which would then be offered to the users. We assume that the developers incurred fixed sunk costs when established as a company for a main activity that is being performed before the IoT platform made its value proposition. The registration for the IoT platform is assumed to cause only incremental revenues and costs, which are the ones relevant for our study and which are described as follows.

Each developer receives an advertising revenue that is dependent on the total number of platform subscribers and its quality as a developer. Specifically, developer *k*’s revenue is equal to $\alpha m^{e}n^{e}q_{k}$, where:

- $m^{e}$ is the number of users that the advertisers expect will subscribe;
- α is the valuation that the advertising agents attach to each subscriber;
- $n^{e}$ is the number of SNs that the advertisers expect will connect;
- $q_{k}$ is a quality factor and is assumed to be a sample of a random variable Q uniformly distributed in the interval $\left[0,1\right]$.

Implicit in the above expression is the fact that the quality of each developer increases proportionally with the number of SNs, i.e., $n^{e}q_{k}$.

On the other hand, each developer should pay an access fee *d* to the platform in order to access the data and to offer its applications.

Developer *k* obtains a profit equal to the revenue $\alpha m^{e}n^{e}q_{k}$ minus the cost *d* if it joins the platform or a profit equal to zero otherwise. We denote the profit expression by ${\hat{\Pi }}_{k}^{d}\equiv \alpha m^{e}n^{e}q_{k}-d$. Thus, the profit of developer *k* is:

(5) $$\Pi_{k}^{d}=\max\left\{0,{\hat{\Pi }}_{k}^{d}\right\},$$

and it will register with the platform if, and only if, ${\hat{\Pi }}_{k}^{d}\geq 0$; and the probability of this event is:

(6) $$P\left(\alpha m^{e}n^{e}Q-d\geq 0\right).$$

The number of registered developers is then a random variable L, and its expected value, *l*, is given by:

(7) $$l=E\;\left[L\right]=LP\left(\alpha m^{e}n^{e}Q-d\geq 0\right).$$

Finally, the developers’ welfare DW is defined as the average aggregate profits of the developers, i.e.,

(8) $$\mathrm{DW}\equiv LE\;\left[\Pi^{d}\right]=L\int_{0}^{1}\max\left\{0,\alpha m^{e}n^{e}q-d\right\}\;dq,$$

which provides a single measure of the overall benefit received by the developers.

### 2.3. Users

Users are interested in accessing a range of sensor-based apps that the platform makes available to them.

Each user pays an access fee *p* to the platform, and this payment entitles the user to download and use apps. We assume that the users’ quality of experience (QoE) depends on the diversity of the developers registered to the platform, since the higher the number of different app developers available through the platform, the more satisfactory the user experience is [15]. Moreover, it also depends on the number of SNs connected to the platform, since the higher the number of SNs supplying data to the platform, the more satisfactory the user experience is.

We denote by $l^{e}$ the number of app developers that the users expect will register. We assume that the users expect that $n^{e}$ SNs will connect, that is that their expectations in this respect are the same as the advertisers’ expectations. The utility that a user *i* receives when subscribing to the platform ${\hat{u}}_{i}$ is then given by:

(9) $${\hat{u}}_{i}=\beta l^{e}n^{e}\omega_{i}-p,$$

where $\omega_{i}$ is the willingness to pay for the QoE of the *i*th user and β is a conversion factor.

Following [16,17], we model a heterogeneous population of *M* users, which are vertically differentiated by their willingness to pay for the QoE of the apps available through the platform. We assume that the willingness to pay is distributed through a random variable Ω uniformly distributed in the interval $\left[0,1\right]$.

Note that we assume that the users are price-takers, which is a sensible assumption for a sufficiently high *M*.

If user *i* does not subscribe to the platform, she/he receives a utility equal to zero. Thus, the utility of user *i* is given as:

(10) $$u_{i}=\max\left\{0,{\hat{u}}_{i}\right\},$$

and she/he will subscribe to the platform if, and only if, ${\hat{u}}_{i}\geq 0$; and the probability of this event is:

(11) $$P\left(\beta l^{e}n^{e}Ω-p\geq 0\right).$$

The number of subscribers M is then a random variable, and its expected value, *m*, is given by:

(12) $$m=E\;\left[M\right]=MP\left(\beta l^{e}n^{e}Ω-p\geq 0\right).$$

Finally, the users’ welfare UW is defined as the average aggregate utility of the users, i.e.,

(13) $$\mathrm{UW}\equiv ME\;\left[u\right]=M\int_{0}^{1}\max\left\{0,\beta l^{e}n^{e}\omega -p\right\}\;d\omega ,$$

which provides a single measure of the overall benefit received by the users.

### 2.4. Equilibrium

We look for fulfilled expectation equilibria [18] where each side’s expectations are fulfilled in (12) and (7)

(14) $$\begin{array}{cc} m^{e} & =m \end{array}$$

(15) $$\begin{array}{cc} l^{e} & =l \end{array}$$

(16) $$\begin{array}{cc} n^{e} & =n. \end{array}$$

An equivalent assumption is that all agents have a perfect foresight [19].

The number of subscribers *m*, registered developers *l*, and connected SNs *n* can be obtained from the solution of (12), (7), and (3), combined with (14)–(16).

### 2.5. The IoT Platform

The platform is assumed to operate as a monopolist, as discussed at the beginning of Section 2. The profit of the platform is given by the revenues from the subscribers and from the registered developers minus the cost incurred in paying the connected SNs, as shown in Figure 2. We assume that other costs are negligible. Therefore, the platform’s expected profits can be expressed as:

(17) $$\Pi^{p}=mp+ld-nf$$

where expressions for *m*, *l*, and *n* can be obtained as described in the previous section.

The platform welfare is equal to its profit, so that we do not use a specific notation. Finally, we define social welfare SW as the sum of the welfare of all stakeholders, i.e.,

(18) $$\mathrm{SW}\equiv \mathrm{UW}+\mathrm{DW}+\mathrm{NW}+\Pi^{p}.$$

## 3. Analysis

Assuming that the monopolistic platform is free to set access fees *p*, *d*, and *f*, the platform faces the problem of choosing *p*, *d*, and *f* to maximize (17). Our interest is to find and characterize:

(19) $$\Pi^{*}=\max_{p,d,f}\;\Pi^{p}>0,$$

and the prices $\left(p^{*},d^{*},f^{*}\right)$ at which the maximum platform profit $\Pi^{*}$ is attained.

In order to determine the domain for (p,d,f) where $\left(p^{*},d^{*},f^{*}\right)$ can exist, we enunciate the following propositions:

**Proposition** **1.** *If $\Pi^{p}>0$, then:*

m>0,l>0,n>0,

*and:*

p<βln,d<αmn,f>0.

**Proof.**  First, we prove by contradiction that neither m=0, nor l=0, nor n=0 is possible if $\Pi^{p}>0$.

- If m=0, ${\hat{\Pi }}_{k}^{d}=-d$, and thus, either d=0 or l=0. Hence, ld=0 and $\Pi^{p}=-nf\leq 0$.
- If l=0, ${\hat{U}}_{i}=-p$, and thus, either p=0 or m=0. Hence, mp=0 and $\Pi^{p}=-nf\leq 0$.
- If n=0, we have both ${\hat{\Pi }}_{k}^{d}=-d$ and ${\hat{U}}_{i}=-p$. Therefore, ld=mp=0, and consequently, $\Pi^{p}=0$.

All three cases lead to a contradiction with the fact that $\Pi^{p}>0$. Consequently, m>0, l>0, and n>0.

Now, from (12), (7), (3), and the fact that m,l,n are positive, it follows that:

(20) $$\begin{array}{cc} P\left(Ω\geq \frac{p}{\beta ln}\right) & >0, \end{array}$$

(21) $$\begin{array}{cc} P\left(Q\geq \frac{d}{\alpha mn}\right) & >0, \end{array}$$

(22) $$\begin{array}{cc} P\left(S\leq \frac{f}{\gamma }\right) & >0. \end{array}$$

Since Ω, Q, and S are uniformly distributed in [0,1], the above inequalities imply p<βln, d<αmn, and f>0. □

**Proposition** **2.** Let us assume that $\Pi^{*}$ exists, and let $\Pi^{p}\left(p^{*},d^{*},f^{*}\right)=\Pi^{*}$, then:

$$p^{*}\geq 0,\;d^{*}\geq 0,\;f^{*}\leq \gamma .$$

**Proof.**  If $p=p^{*}<0$, ${\hat{U}}_{i}=\beta ln\omega -p^{*}>0$. Thus, all users will subscribe to the platform: m=M>0. This allows us to write:

(23) $$\Pi^{p}{|}_{p=p^{*}}=Mp^{*}+\Pi^{p}{|}_{p=0}<\Pi^{p}{|}_{p=0},$$

which contradicts our assumption that the maximum is attained when $p=p^{*}$.

Similarly, $d=d^{*}<0$ leads to the contradiction:

(24) $$\Pi^{p}{|}_{d=d^{*}}=Ld^{*}+\Pi^{p}{|}_{d=0}<\Pi^{p}{|}_{d=0}.$$

Finally, if $f=f^{*}>\gamma$, n=N, and we obtain the contradiction:

(25) $$\Pi^{p}{|}_{f=f^{*}}=\Pi^{p}{|}_{f=\gamma }-N\left(f-\gamma \right)<\Pi^{p}{|}_{f=\gamma }.$$

□

Now, since we are interested in finding $\Pi^{*}>0$, we can restrict the variation of prices as:

(26) $$\begin{array}{cc} p & \in [0,\beta ln) \end{array}$$

(27) $$\begin{array}{cc} d & \in [0,\alpha mn) \end{array}$$

(28) $$\begin{array}{cc} f & \in (0,\gamma ]. \end{array}$$

With these restrictions, the expressions for *m*, *l*, and *n* (given in (12), (7), and (3)) become:

(29) $$\begin{array}{cc} m & =M\left(1-\frac{p}{\beta ln}\right), \end{array}$$

(30) $$\begin{array}{cc} l & =L\left(1-\frac{d}{\alpha mn}\right), \end{array}$$

(31) $$\begin{array}{cc} n & =N\frac{f}{\gamma }, \end{array}$$

and from these, we obtain the prices as functions of *m*, *l*, and *n*:

(32) $$\begin{array}{cc} p & =\beta ln\left(1-\frac{m}{M}\right), \end{array}$$

(33) $$\begin{array}{cc} d & =\alpha mn\left(1-\frac{l}{L}\right), \end{array}$$

(34) $$\begin{array}{cc} f & =\gamma \frac{n}{N}. \end{array}$$

Now, substituting the expressions of *p*, *d*, and *f* into $\Pi^{p}$ gives:

(35) $$\Pi^{p}\left(m,l,n\right)=mln\left(\alpha +\beta -\alpha \frac{l}{L}-\beta \frac{m}{M}\right)-\gamma N{\left(\frac{n}{N}\right)}^{2}.$$

Moreover, from (4), (8), and (13), it easily follows that:

(36) $$\begin{array}{cc} \mathrm{NW} & =N\frac{f^{2}}{2\gamma }, \end{array}$$

(37) $$\begin{array}{cc} \mathrm{DW} & =L\frac{\alpha mn}{2}{\left(1-\frac{d}{\alpha mn}\right)}^{2}, \end{array}$$

(38) $$\begin{array}{cc} \mathrm{UW} & =M\frac{\beta ln}{2}{\left(1-\frac{p}{\beta ln}\right)}^{2}. \end{array}$$

We note that the expression for $\Pi^{p}\left(m,l,n\right)$ given in (35) also yields a valid value when m=0, l=0, or n=0 (see the proof of Proposition 1).

Thus, $\Pi^{p}\left(m,l,n\right)$ is defined in [0,M]×[0,L]×[0,N], which is a compact set (closed and bounded), and is continuous (and differentiable) in this domain. This guarantees that $\Pi^{p}\left(m,l,n\right)$ attains a maximum, $\Pi^{*}$, in [0,M]×[0,L]×[0,N]. Furthermore, since we are interested in the case $\Pi^{*}>0$, the maximum must be attained in (0,M]×(0,L]×(0,N]. The problem of maximization is then formulated as:

(39) $$\begin{array}{ccc} \mathrm{Maximize} & \Pi^{p}\left(m,l,n\right) & \\ \mathrm{subject}\;\mathrm{to} & m,l,n>0, & \\ & g_{1}\left(m,l,n\right)=m-M & \leq 0,\\ & g_{2}\left(m,l,n\right)=l-L & \leq 0,\\ & g_{3}\left(m,l,n\right)=n-N & \leq 0. \end{array}$$

First, we find the points (m,l,n) in [0,M]×[0,L]×[0,N] that meet the necessary conditions for a local maximum. As shown below, for all possible values of M,L,N,α,β,γ>0, there exists one, and only one, solution to the necessary conditions, which is denoted by $\left(m^{*},l^{*},n^{*}\right)$. Therefore, since the continuity of $\Pi^{p}\left(m,l,n\right)$ ensures the existence of a global maximum in [0,M]×[0,L]×[0,N], we conclude that a global maximum is attained at $\left(m^{*},l^{*},n^{*}\right)$. Moreover, it is also shown that $\Pi^{*}=\Pi^{p}\left(m^{*},l^{*},n^{*}\right)>0$.

The necessary conditions are given by the Karush–Kuhn–Tucker (KKT) conditions:

(40) $$\begin{array}{cc} \nabla \Pi^{p}\left(m,l,n\right) & =\mu_{1}\nabla g_{1}+\mu_{2}\nabla g_{2}+\mu_{3}\nabla g_{3}\\ & =\mu_{1}{\left[1\;0\;0\right]}^{T}+\mu_{2}{\left[0\;1\;0\right]}^{T}+\mu_{3}{\left[0\;0\;1\right]}^{T}\\ & ={\left[\mu_{1}\;\mu_{2}\;\mu_{3}\right]}^{T},\\ \mu_{1},\mu_{2},\mu_{3} & \geq 0,\\ \mu_{1}\left(m-M\right) & =0,\\ \mu_{2}\left(l-L\right) & =0,\\ \mu_{3}\left(n-N\right) & =0, \end{array}$$

which, applied to the expression of $\Pi^{p}$ given in (35), can be written as:

(41) $$\begin{array}{ccc} \frac{\partial \Pi^{p}}{\partial m} & =ln\left(\alpha +\beta -\alpha \frac{l}{L}-\beta \frac{m}{M}\right)-\beta \frac{m}{M}ln & =\mu_{1}\geq 0, \end{array}$$

(42) $$\begin{array}{ccc} \frac{\partial \Pi^{p}}{\partial l} & =mn\left(\alpha +\beta -\alpha \frac{l}{L}-\beta \frac{l}{L}\right)-\alpha \frac{l}{L}mn & =\mu_{2}\geq 0, \end{array}$$

(43) $$\begin{array}{ccc} \frac{\partial \Pi^{p}}{\partial n} & =ln\left(\alpha +\beta -\alpha \frac{l}{L}-\beta \frac{m}{M}\right)-2\gamma \frac{n}{N} & =\mu_{3}\geq 0, \end{array}$$

and:

(44) $$\begin{array}{cc} \mathrm{if}\;m<M,\; & \mathrm{then}\;\mu_{1}=0, \end{array}$$

(45) $$\begin{array}{cc} \mathrm{if}\;l<L,\; & \mathrm{then}\;\mu_{2}=0, \end{array}$$

(46) $$\begin{array}{cc} \mathrm{if}\;n<N,\; & \mathrm{then}\;\mu_{3}=0. \end{array}$$

We will study separately the types of possible solutions for each of the following regions of the $\Pi^{p}\left(m,l,n\right)$ domain:

region
$m^{*}$

$l^{*}$

$n^{*}$

$R_{MLN}$

=M

=L

=N

$R_{MLn}$

=M

=L

<N

$R_{MlN}$

=M

<L

=N

$R_{Mln}$

=M

<L

<N

$R_{mLN}$

<M

=L

=N

$R_{mLn}$

<M

=L

<N

$R_{mlN}$

<M

<L

=N

$R_{mln}$

<M

<L

<N


### 3.1. Regions $R_{MLN}$ and $R_{MLn}$ ($m^{*}=M$, $l^{*}=L$)

By substituting $m^{*}=M$ and $l^{*}=L$ in (41), we see that the solutions in $R_{MLN}$ and $R_{MLn}$ are not feasible.

### 3.2. Regions $R_{MlN}$ and $R_{Mln}$ ($m^{*}=M$, $l^{*}<L$)

By substituting m=M in (42) with $\mu_{2}=0$, we obtain that:

(47) $$l^{*}=\frac{L}{2},$$

and by substituting m=M and l=L/2 in (41), we see that this solution exists when:

(48) $$\frac{\alpha }{\beta }\geq 2.$$

To check if this solution exists for $n^{*}=N$, we substitute m=M, l=L/2, and n=N in (43), and we see that it does if:

(49) $$\frac{\gamma }{\beta }\leq \frac{ML}{8}\frac{\alpha }{\beta }.$$

Therefore, if the above condition is met, (M,L/2,N) is a maximum in (0,M]×(0,L]×(0,N], and:

(50) $$\Pi^{*}=\gamma N\left(\frac{\alpha }{4\gamma }ML-1\right)>0.$$

The solution for $n^{*}<N$ is found by substituting m=M and l=L/2 in (43) with $\mu_{3}=0$, resulting in:

(51) $$n^{*}=\frac{MLN}{8}\frac{\alpha }{\gamma },$$

which exists if:

(52) $$\frac{\gamma }{\beta }>\frac{ML}{8}\frac{\alpha }{\beta }.$$

This solution is also a maximum in (0,M]×(0,L]×(0,N], and:

(53) $$\Pi^{*}=\gamma N{\left(\frac{n^{*}}{N}\right)}^{2}>0.$$

### 3.3. Regions $R_{mLN}$ and $R_{mLn}$ ($m^{*}<M$, $l^{*}=L$)

Proceeding in an analogous way to the previous case, we obtain that the solution is $m^{*}=M/2$, and it exists if:

(54) $$\frac{\alpha }{\beta }\leq 2.$$

For $n^{*}=N$, the solution exists if:

(55) $$\frac{\gamma }{\beta }\leq \frac{ML}{8},$$

and:

(56) $$\Pi^{*}=\gamma N\left(\frac{\beta }{4\gamma }ML-1\right)>0.$$

For $n^{*}<N$, the solution is $n^{*}=MLN\left(\beta /8\gamma \right)$, and it exists if:

(57) $$\frac{\gamma }{\beta }>\frac{ML}{8},$$

and:

(58) $$\Pi^{*}=\gamma N{\left(\frac{n^{*}}{N}\right)}^{2}>0.$$

### 3.4. Regions $R_{mlN}$ and $R_{mln}$ ($m^{*}<M$, $l^{*}<L$)

By solving the system formed by (41) with $\mu_{1}=0$ and (42) with $\mu_{2}=0$, we obtain that:

(59) $$\begin{array}{c} m^{*}=\frac{\alpha +\beta }{3\beta }M, \end{array}$$

(60) $$\begin{array}{c} l^{*}=\frac{\alpha +\beta }{3\alpha }L, \end{array}$$

which exist for $m^{*}<M$ and $l^{*}<L$ if:

(61) $$\frac{1}{2}<\frac{\alpha }{\beta }<2.$$

To check this solution for $n^{*}=N$, we substitute the values of *m* and *l* given by (59) and (60), and n=N, in (43), and we find that it exists if:

(62) $$\frac{\gamma }{\beta }\leq \frac{ML}{2\frac{\alpha }{\beta }}{\left(\frac{1+\frac{\alpha }{\beta }}{3}\right)}^{3};$$

it results in:

(63) $$\Pi^{*}=\gamma N\left({\left(\frac{\alpha +\beta }{3}\right)}^{3}\frac{ML}{\alpha \beta \gamma }-1\right)>0.$$

The solution for $n^{*}<N$ is found by substituting the values of *m* and *l* given by (59) and (60) in (43) with $\mu_{3}=0$, resulting in:

(64) $$n^{*}=\frac{ML}{2\alpha \beta \gamma }{\left(\frac{\alpha +\beta }{3}\right)}^{3}N,$$

which exists if:

(65) $$\frac{\gamma }{\beta }>\frac{ML}{2\frac{\alpha }{\beta }}{\left(\frac{1+\frac{\alpha }{\beta }}{3}\right)}^{3},$$

and results in:

(66) $$\Pi^{*}=\gamma N{\left(\frac{n^{*}}{N}\right)}^{2}>0.$$

### 3.5. Summary of the Results of the Analysis

**Figure 3:**
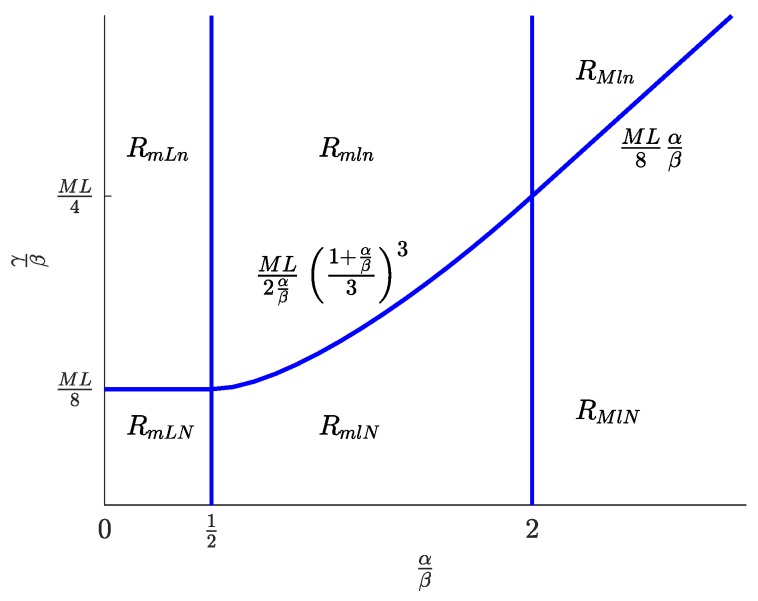
Regions of existence of the different solution types.

Figure 3 displays the regions where each of the solution types exists. The limits of these regions are given by the values of the parameters α, β, and γ. Parameter α is the valuation that the advertising agents attach to each subscriber; parameter β is the coefficient of proportionality between the user utility and the product of the number of connected SNs and the number of registered developers; and parameter γ is the price per upload capacity unit paid by each SN. In Figure 3, the regions are represented in a plane defined by α and γ normalized to β. As seen, in the α/β axis, the regions are simply bounded by the thresholds 1/2 and two, while in the γ/β axis, there is a single border that is a piecewise function defined by (55), (62), and (49).

Table 2 contains the expressions of $m^{*}$, $l^{*}$, and $n^{*}$ for the six solution types. Knowing these values, optimal prices $p^{*}$, $d^{*}$, and $f^{*}$ can be obtained from (32)–(34), and the welfare of SNs, developers, and users can be obtained from (36)–(38), while the platform profit can be obtained from (17).

### 3.6. Price Elasticity of Demands

Here, we analyze the price elasticity of the demands *m* and *l*, defined as:

(67) $$\begin{array}{cc} E_{m,p} & \equiv \frac{\partial m}{\partial p}\frac{p}{m}, \end{array}$$

(68) $$\begin{array}{cc} E_{l,d} & \equiv \frac{\partial l}{\partial d}\frac{d}{l}, \end{array}$$

in the equilibrium $\left(m^{*},l^{*},n^{*}\right)$.

Taking derivatives in (29)–(31), we obtain:

(69) $$\begin{array}{cc} \frac{\partial m}{\partial p} & =\frac{M}{\beta ln}{\left(\frac{M}{m}\frac{p}{\beta ln}\frac{L}{l}\frac{d}{\alpha mn}-1\right)}^{-1} \end{array}$$

(70) $$\begin{array}{cc} \frac{\partial l}{\partial d} & =\frac{L}{\alpha mn}{\left(\frac{M}{m}\frac{p}{\beta ln}\frac{L}{l}\frac{d}{\alpha mn}-1\right)}^{-1} \end{array}$$

Now, using (32)–(33), we can write:

(71) $$\begin{array}{cc} E_{m,p} & =\frac{\frac{M}{m}-1}{\left(\frac{M}{m}-1\right)\left(\frac{L}{l}-1\right)-1}, \end{array}$$

(72) $$\begin{array}{cc} E_{l,d} & =\frac{\frac{L}{l}-1}{\left(\frac{M}{m}-1\right)\left(\frac{L}{l}-1\right)-1}. \end{array}$$

Finally, substituting the values of $m^{*}$ and $l^{*}$ from Table 2 into (71) and (72) yields the elasticities for each equilibrium type as summarized in Table 3. Note the following characteristics of the elasticities:

- the expressions of the two elasticities are independent of the SNs’ side, that is they are not dependent on either *n*, or γ, or *f*;
- the absolute values are less than or equal to one, that is the demands at the users’ and developers’ sides are inelastic;
- a higher relative valuation α/β translates into a less elastic user demand and a more elastic developer demand.

## 4. Results and Discussion

In this section, we present and discuss some numerical results to illustrate the main characteristics of the model.

### 4.1. Optimum Prices and Equilibrium Values

**Figure 4:**
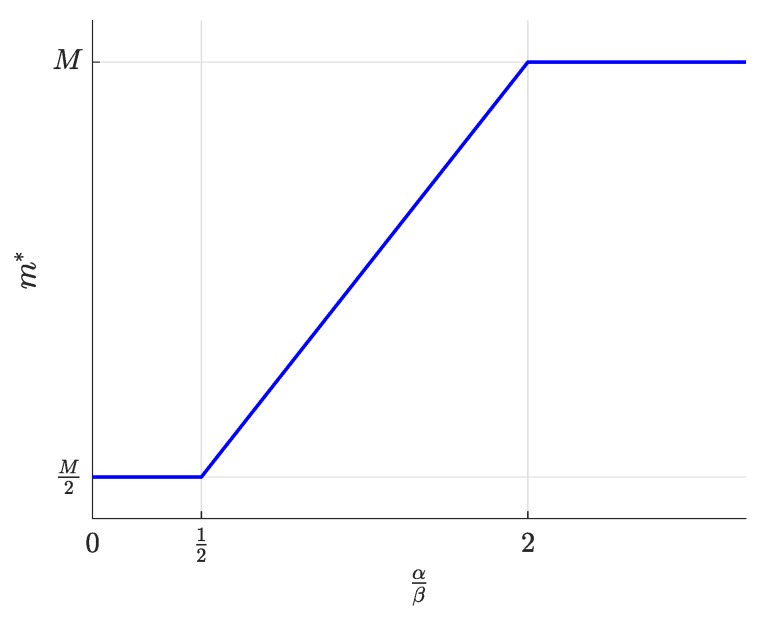
Number of subscribers $m^{*}$ as a function of the normalized advertiser valuation $\frac{\alpha }{\beta }$.

**Figure 5:**
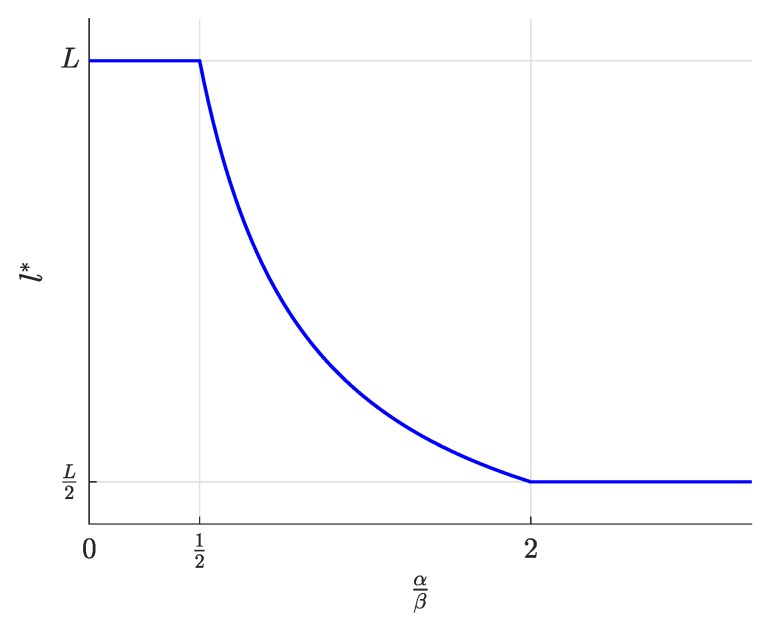
Number of registered developers $l^{*}$ as a function of the normalized advertiser valuation $\frac{\alpha }{\beta }$.

**Figure 6:**
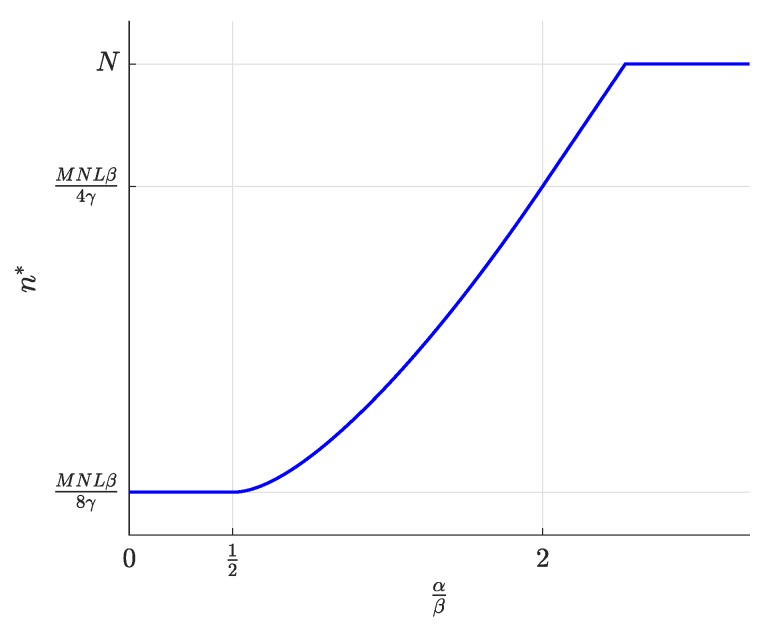
Number of connected SNs $n^{*}$ as a function of the normalized advertiser valuation $\frac{\alpha }{\beta }$.

**Figure 7:**
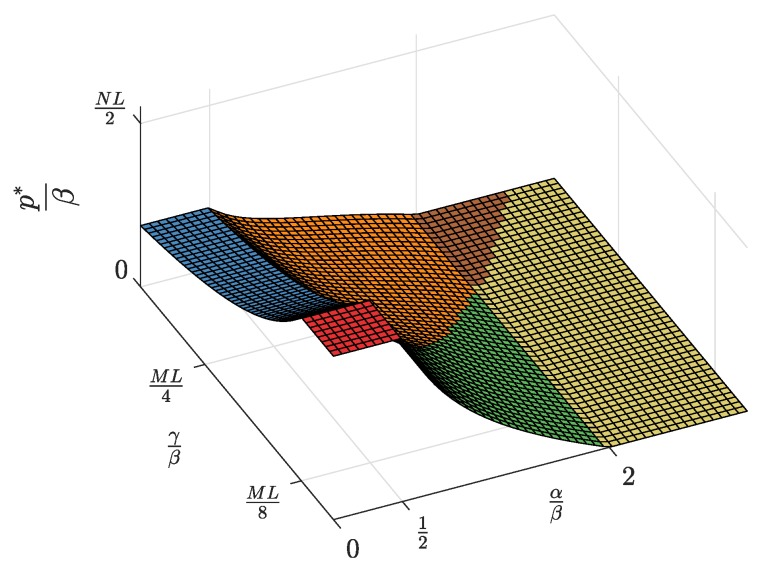
Normalized subscription price $p^{*}$ as a function of the normalized advertiser valuation $\frac{\alpha }{\beta }$ and the normalized network access fee $\frac{\gamma }{\beta }$.

**Figure 8:**
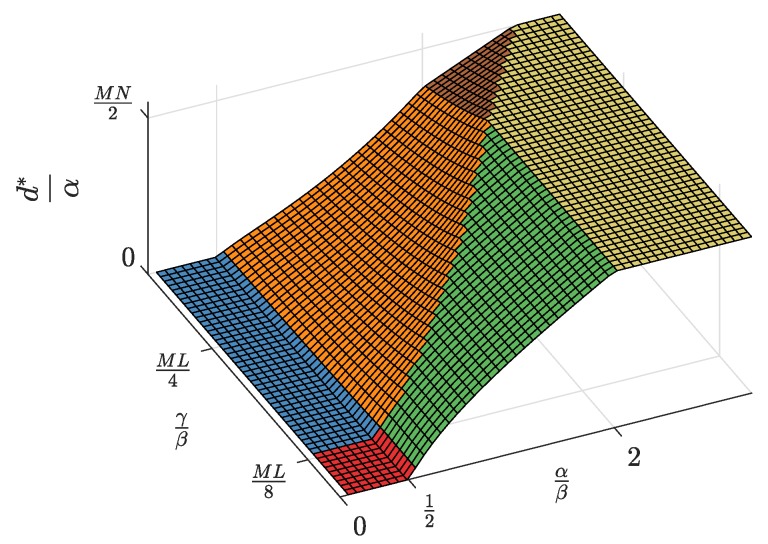
Normalized access fee $d^{*}$ as a function of the normalized advertiser valuation $\frac{\alpha }{\beta }$ and the normalized network access fee $\frac{\gamma }{\beta }$.

**Figure 9:**
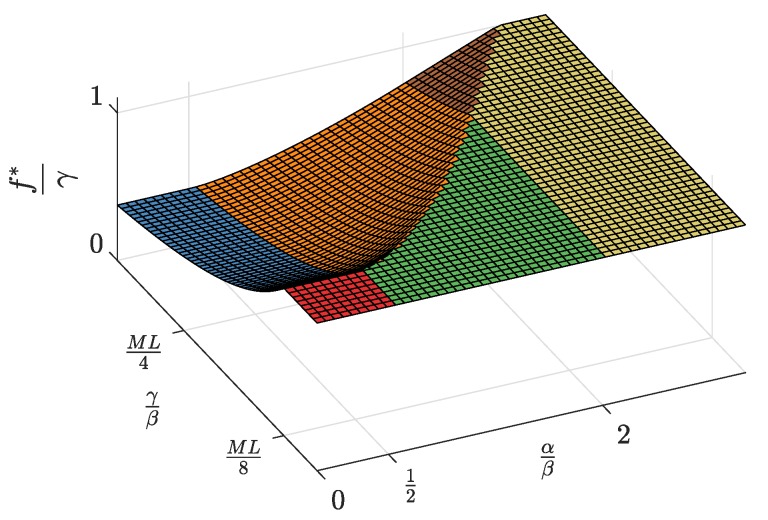
Normalized SN payment $f^{*}$ as a function of the normalized advertiser valuation $\frac{\alpha }{\beta }$ and the normalized network access fee $\frac{\gamma }{\beta }$.

First, the equilibrium values $m^{*}$, $l^{*}$, and $n^{*}$, whose expressions were previously shown in Table 2, are now represented in Figure 4, Figure 5 and Figure 6 and are discussed together with the optimum prices $p^{*}$, $d^{*}$, and $f^{*}$, which are represented as normalized values in Figure 7, Figure 8 and Figure 9.

First, we focus on the dependence of the optimum prices and the equilibrium values on α/β.

- For α/β>2, the maximum profits are achieved when the users are offered a free service ($p^{*}=0$, Figure 7), and therefore, all users subscribe to the service ($m^{*}=M$, Figure 4). The users’ subscription is then subsidized by the advertisers. Note that a high value of α means that a subscriber is highly valued by the agents willing to advertise through the apps. On the other hand, half of the developers register (l=L/2, Figure 5). Finally, the number of connected SNs, $n^{*}$, also reaches the maximum value *N* (Figure 6), which is achieved when $f^{*}$ reaches the value γ (Figure 9). The platform does not need to pay a fee higher than this value. This highest payment occurs for α/β greater than a threshold that depends on the value of the other parameters.
- For α/β<1/2, maximum profits are instead achieved when all developers register for free ($l^{*}=L$ and $d^{*}=0$, Figure 5 and Figure 8, respectively). In this case, the subscribers are charged a non-zero price (Figure 7). As a kind of dual situation compared to the previous case, half of the users subscribe ($m^{*}=M/2$, Figure 4). The developers’ registration is now subsidized by the subscribers. As regards the SNs, they are paid the lowest fee (check the surface cut for a fixed γ/β in Figure 9), and the number of connected SNs is correspondingly at the lowest value (Figure 6).
- For intermediate values of α/β, a fraction of the users subscribe and a fraction of the developers register, both paying non-zero fees. Moreover, a fraction of the SNs connects and receives a non-zero fee. Recalling the computation of the price elasticities $E_{m,p}$ and $E_{l,d}$ made in Section 3.6, we note that as α/β moves from 1/2 to two, the demand $m^{*}$ becomes more inelastic, that is an eventual $p^{*}$ reduction has less impact on the subscription, which approaches 100% (Figure 4). Conversely, as α/β moves from two to 1/2, the demand $l^{*}$ becomes more inelastic, that is an eventual $d^{*}$ reduction has less impact on the developers’ registration, which approaches 100% (Figure 5).

Second, it can be checked that the above discussion is qualitatively the same for any value of γ/β for $m^{*}$, $l^{*}$, $p^{*}$, and $d^{*}$. In contrast, for $n^{*}$ and $f^{*}$, there is a qualitative difference when γ/β goes below ML/8: $n^{*}$ is *N* for all α/β (Figure 6, when MNLβ/8γ reaches *N*), and $f^{*}$ is also constant and equal to γ (Figure 9).

And third, $n^{*}$ is proportional to ML for the intermediate range of values of α/β (check Table 2), so that there is an incentive for the SNs to join the platform if the population size from either of the other two sides increases in the long term. Otherwise, there is only the dependence of $m^{*}$, $l^{*}$, and $n^{*}$ on their respective population sizes *M*, *L*, and *N*, so that no other cross influence prevails in the long term. Nevertheless, our analysis is static, and these sorts of dynamic considerations are qualitative comparative statics.

### 4.2. Welfare

Next, we proceed to discuss the welfare of every group of agents (users, developers, SNs, and platform) when the platform fixes the optimum prices for the three sides. we want to discuss the effect of *M*, *L*, *N*, and α.

**Figure 10:**
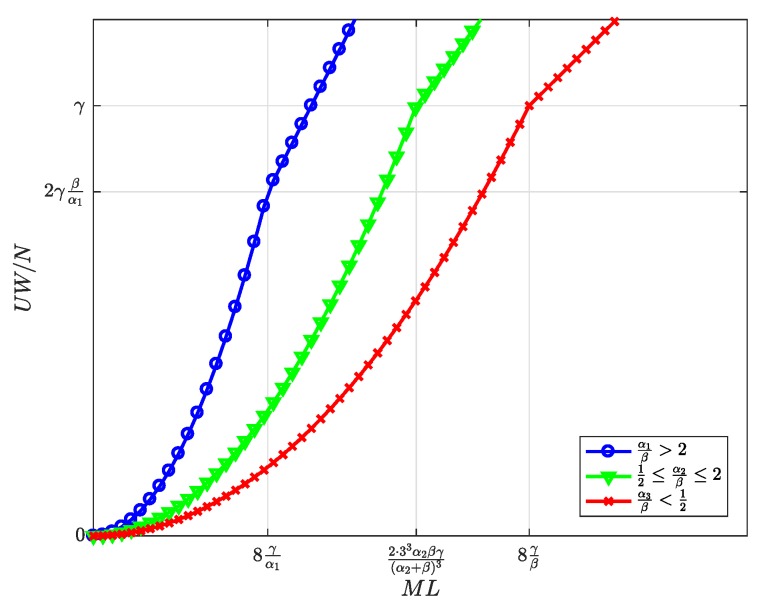
Normalized users’ welfare at the optimum as a function of the user-developer population product ML.

**Figure 11:**
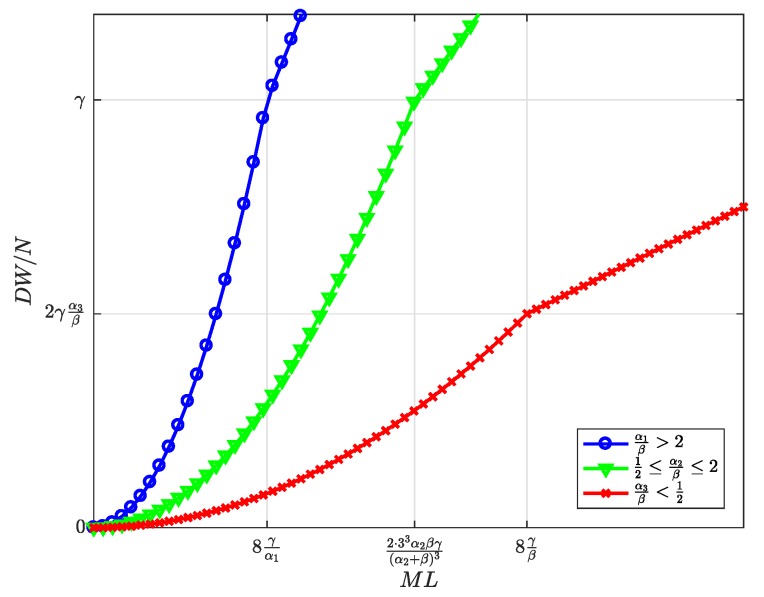
Normalized developers’ welfare at the optimum as a function of the user-developer population product ML.

**Figure 12:**
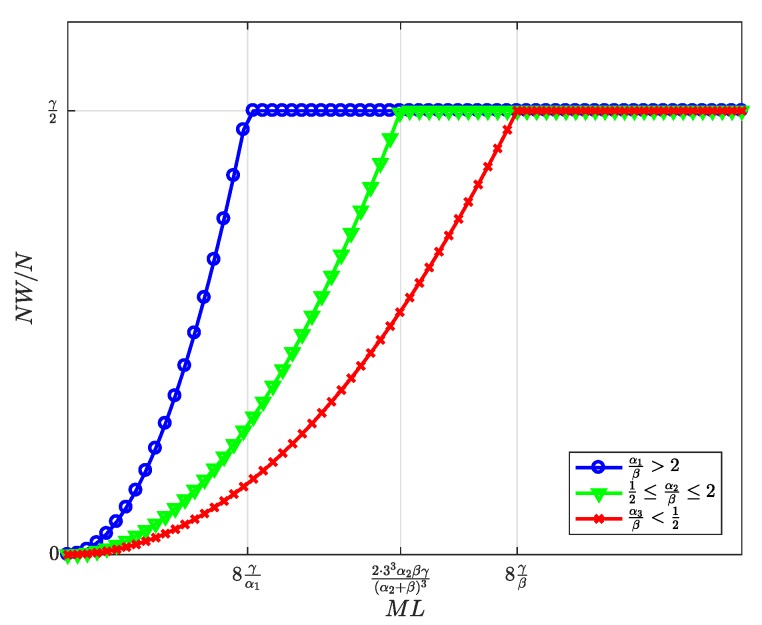
Normalized SNs’ welfare at the optimum as a function of the user-developer population product ML.

**Figure 13:**
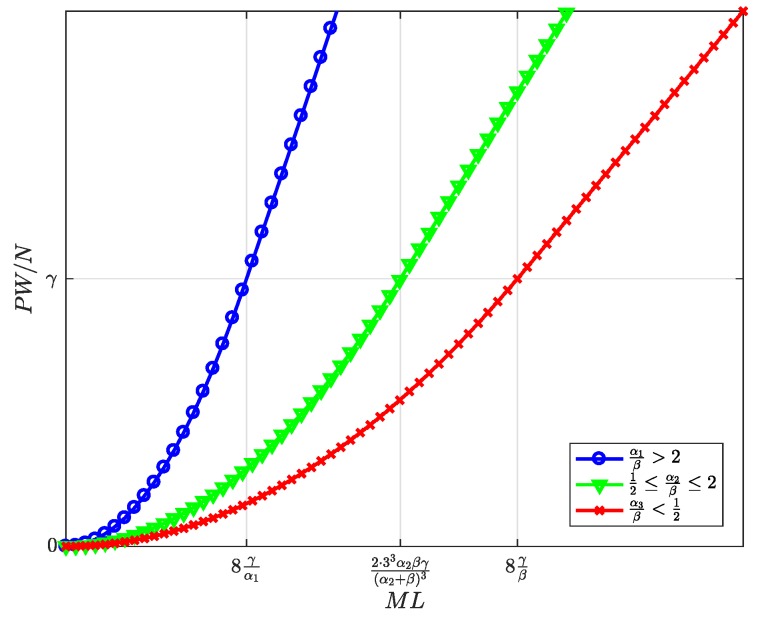
Normalized maximum platform’s profit as a function of the user-developer population product ML.

**Figure 14:**
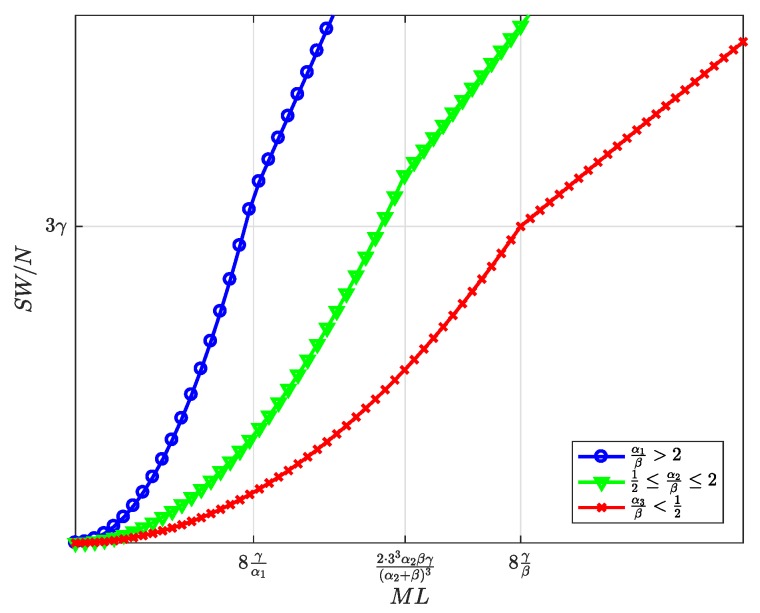
Normalized social welfare at the optimum as a function of the user-developer population product ML.

Figure 10, Figure 11, Figure 12, Figure 13 and Figure 14 represent the welfare expressions, normalized to *N*, as a function of the user-developer population product, ML, for three different ranges of the value of α/β. The normalization is motivated by the fact that the expressions are proportional to *N*.

First, it should be highlighted that all welfare is non-decreasing with ML. As regards UW and DW, they increase first quadratically and later linearly. This implies that whenever *L* increases, the increase in $l^{*}$ (Figure 5) compensates for the increase in the price $p^{*}$ for the users (Figure 7), and this increases UW (Figure 10). Alternatively, whenever *M* increases, the increase in the number of subscribers $m^{*}$ (Figure 4) compensates for the increase in the price $d^{*}$ for the developers (Figure 8), and this increases DW (Figure 11). NW increases first quadratically and then keeps constant (Figure 12), once all SNs are connected (Figure 6), and therefore, the fee $\gamma^{*}$ does not need to increase beyond γ (Figure 9). PW increases quadratically for all ML (Figure 13). Furthermore, SW increases with ML (Figure 14), consequently.

The positive influence that *M*, *L*, and *N* exert over the welfare of every group of agents can provide a rationale for increasing the user population (*M*), e.g., through marketing campaigns or subsidies. However, it can also provide a business case for the entry of enterprises specialized in developing sensor-based applications (*L*) and for the deployment of sensor networks (*N*).

Second, all welfare is proportional to *N*. Indeed, higher values of *N* drive SNs’ participation up (Figure 6), which explains the increase in NW. Moreover, higher values of *N* also drive $p^{*}$ and $d^{*}$ up, which explains the increase in PW. Furthermore, despite the increase in the price $p^{*}$ paid by the subscribers and the price $d^{*}$ paid by the developers, we observe that UW increases and that DW also increases with respect to *N*.

Finally, higher values of α/β drive all groups’ welfare up. As regards the users, this implies that the decrease in the price $p^{*}$ (Figure 7) compensates for the decrease in the number of registered developers $l^{*}$ (Figure 8). As regards the developers, this implies that the increase in the number of subscribers $m^{*}$ (Figure 4) compensates for the increase in the price $d^{*}$ (Figure 8).

**Figure 15:**
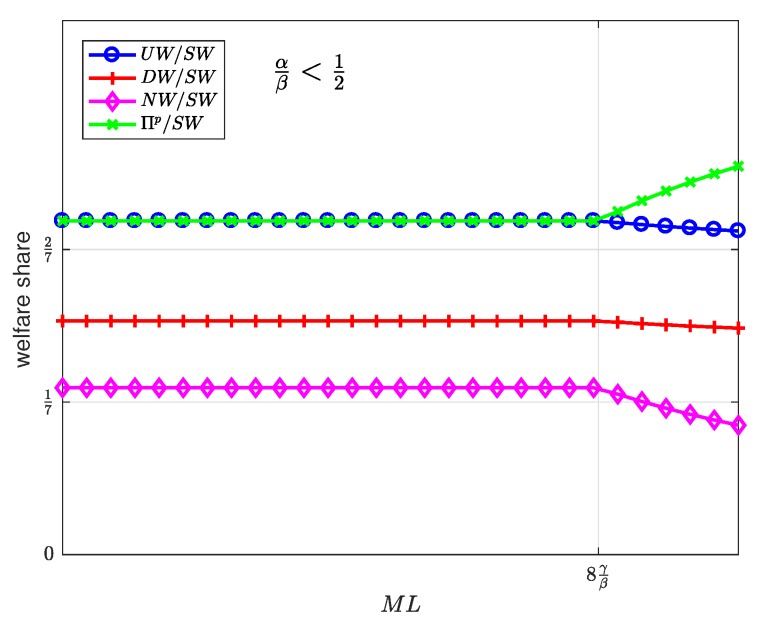
Welfare share as a function of the user-developer population product ML for a normalized advertiser valuation $\frac{\alpha }{\beta }<\frac{1}{2}$.

**Figure 16:**
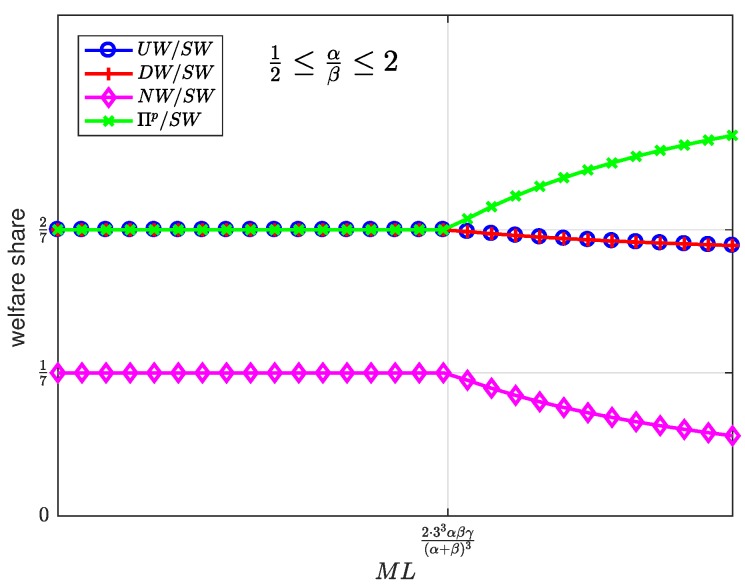
Welfare share as a function of the user-developer population product ML for a normalized advertiser valuation $\frac{1}{2}\leq \frac{\alpha }{\beta }\leq 2$.

**Figure 17:**
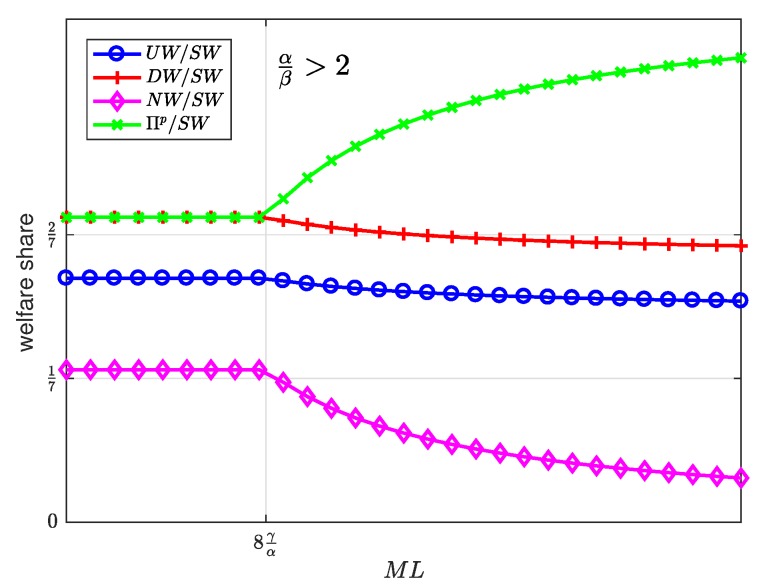
Welfare share as a function of the user-developer population product ML for a normalized advertiser valuation $2<\frac{\alpha }{\beta }$.

Figure 15, Figure 16 and Figure 17 show the welfare distribution among the groups of agents as a function of ML for three ranges of the value of α/β.

It can be observed that the platform always gets the largest share of the welfare, while the SNs get the smallest share.

The welfare distributes in an egalitarian manner between some groups of agents as long as ML is below a threshold value. Specifically, the welfare is equally shared between the users and the platform (Figure 15); between the users, the platform and the developers (Figure 16); or between the platform and the developers (Figure 17). Beyond the threshold value, which depends on the values of α, β, and γ, the welfare distribution is increasingly inequitable. This observation allows us to conclude, first, that the intermediate region $\alpha /\beta \in \left[1/2,2\right]$ exhibits the most egalitarian welfare distribution, at the expense of the platform share; and second, that moderate values of the product ML maintain an egalitarian welfare distribution, regardless of the value of α/β.

To summarize the above discussion: every welfare is directly proportional to *N* and non-decreasing in ML and is distributed in an egalitarian manner between the users, the platform, and the developers for intermediate values of α/β and moderate values of ML.

## 5. Conclusions

A business model for the provision of sensor-based services has been proposed. This business model exhibits a platform aiming to create a multi-sided market where users, SNs, and app developers interact and are charged accordingly by the platform. A scenario where only a platform is present in the market is analyzed, and the effect of some parameters is computed and discussed.

We have shown first that profit maximizing platform prices exist for any parameter values and that these prices always induce an equilibrium in the number of agents from each group that join the platform.

Second, we have shown that the relative strength of the value that advertisers attach to the subscribers, the ratio $\frac{\alpha }{\beta }$, determines the platform price structure. Specifically, two alternative subsidizing strategies are feasible: to subsidize either the users’ subscription (when that value is relatively strong, i.e., $\frac{\alpha }{\beta }>2$) or the developers’ registration (when it is relatively weak, i.e., $\frac{\alpha }{\beta }<\frac{1}{2}$).

All in all, a high normalized advertiser valuation is beneficial for all groups of agents (users, platform, developers, and SNs), as the welfare computation in Section 4.2 has shown. However, there is a welfare distribution effect that must be considered, since intermediate normalized advertiser valuations $\frac{1}{2}\leq \frac{\alpha }{\beta }\leq 2$ exhibit the most egalitarian welfare distribution between the groups of agents.

Finally, all groups of agents benefit from an increase in the population at any of the three sides, and this fact would provide a rationale for incentivizing not only the user participation, i.e., high values of *M*, but also the entry of developer undertakings, i.e., high values of *L*, and the deployment of SN infrastructure, i.e., high values of *N*.

From a practical perspective, this analysis provides a basic rationale for the deployment of a platform for an Internet-of-Things market supported by a feasible business model. The proposed business model intends to translate the success stories from the mobile app arena to the IoT arena and provides some operating guidance in terms of which side to incentivize. Finally, our proposal may be adopted or taken into account by the current emerging IoT platforms.

## Figures and Tables

**Table 1:** Summary of notation.

		Equation	Page
**System Parameters**			
Number of users	*M*		3
Number of developers	*L*		3
Number of sensor networks (SNs)	*N*		3
Conversion factor	β		7
Valuation of a subscriber	α		6
Network access fee per upload capacity unit	γ		5
**Optimization Parameters**			
Fee paid by each user to the platform	*p*		7
Fee paid by each developer to the platform	*d*		6
Fee paid by the platform to each SN	*f*		5
Values of *p*, *d*, and *f* at which the maximum platform’s profits are attained	$p^{*},d^{*},f^{*}$		8
**Random Variables**			
Number of subscribers	M		7
Number of registered developers	L		7
Number of connected SNs	N		6
User willingness to pay	Ω		7
Developer quality factor	Q		6
SN upload requirements	S		6
Expected number of M	*m*	(12)	7
Expected value of L	*l*	(7)	7
Expected value of N	*n*	(3)	6
Value of Ω for user *i*	$w_{i}$		7
Value of Q for developer *k*	$q_{k}$		6
Value of S for SN *j*	$s_{j}$		5
Number of users that the advertisers expect will subscribe	$m^{e}$		6
Number of developers that the users expect will register	$l^{e}$		7
Number of SNs that the advertisers expect will connect	$n^{e}$		6
**Profits and Welfare**			
Utility of user *i*	$u_{i}$	(10)	7
Profits of developer *k*	$\Pi_{k}^{d}$	(5)	6
Profits of SN *i*	$\Pi_{i}^{s}$	(1)	6
Platform’s profits	$\Pi^{p}$	(17)	8
Maximum platform’s profits	$\Pi^{*}$	(19)	8
Users’ welfare	UW	(13)	7
Developers’ welfare	DW	(8)	7
SNs’ welfare	NW	(4)	6
Social welfare	SW	(18)	8

**Table 2:** Summary of the results of the analysis.

Region	$m^{*}$	$l^{*}$	$n^{*}$
$R_{MlN}$ $R_{Mln}$	*M*	$\frac{L}{2}$	*N* $\frac{\alpha }{8\gamma }MNL$
$R_{mLN}$ $R_{mLn}$	$\frac{M}{2}$	*L*	*N* $\frac{\beta }{8\gamma }MNL$
$R_{mlN}$ $R_{mln}$	$\frac{\alpha +\beta }{3\beta }M$	$\frac{\alpha +\beta }{3\alpha }L$	*N* $\frac{MLN}{2\alpha \beta \gamma }{\left(\frac{\alpha +\beta }{3}\right)}^{3}$

**Table 3:** Price elasticity of demands in the equilibrium.

	Regions	$E_{m,p}\left(m^{*},l^{*},n^{*}\right)$	$E_{l,d}\left(m^{*},l^{*},n^{*}\right)$
$\frac{\alpha }{\beta }\leq \frac{1}{2}$	$R_{mLN},R_{mLn}$	−1	0
$\frac{1}{2}\leq \frac{\alpha }{\beta }\leq 2$	$R_{mlN},R_{mln}$	$\frac{1}{3}-\frac{1}{{\left(\alpha /\beta \right)}^{2}-\alpha /\beta +1}$	$\frac{1}{3}-\frac{1}{{\left(\alpha /\beta \right)}^{-2}-{\left(\alpha /\beta \right)}^{-1}+1}$
$2\leq \frac{\alpha }{\beta }$	$R_{MlN},R_{Mln}$	0	−1
